# Reversing Post-Infectious Epigenetic-Mediated Immune Suppression

**DOI:** 10.3389/fimmu.2021.688132

**Published:** 2021-06-07

**Authors:** Abhimanyu, Carlos O. Ontiveros, Rosa S. Guerra-Resendez, Tomoki Nishiguchi, Malik Ladki, Isaac B. Hilton, Larry S. Schlesinger, Andrew R. DiNardo

**Affiliations:** ^1^ The Global Tuberculosis Program, William T. Shearer Center for Human Immunobiology, Texas Children’s Hospital, Immigrant and Global Health, Baylor College of Medicine, Houston, TX, United States; ^2^ Host-Pathogen Interactions Program, Texas Biomedical Research Institute, San Antonio, TX, United States; ^3^ UT Health San Antonio, San Antonio, TX, United States; ^4^ Systems, Synthetic, and Physical Biology Graduate Program, Rice University, Houston, TX, United States; ^5^ Department of Bioengineering, Rice University, Houston, TX, United States; ^6^ Department of BioSciences, Rice University, Houston, TX, United States

**Keywords:** tolerance, immune exhaustion, epigenetics, chronic infections, bioengineering

## Abstract

The immune response must balance the pro-inflammatory, cell-mediated cytotoxicity with the anti-inflammatory and wound repair response. Epigenetic mechanisms mediate this balance and limit host immunity from inducing exuberant collateral damage to host tissue after severe and chronic infections. However, following treatment for these infections, including sepsis, pneumonia, hepatitis B, hepatitis C, HIV, tuberculosis (TB) or schistosomiasis, detrimental epigenetic scars persist, and result in long-lasting immune suppression. This is hypothesized to be one of the contributing mechanisms explaining why survivors of infection have increased all-cause mortality and increased rates of unrelated secondary infections. The mechanisms that induce epigenetic-mediated immune suppression have been demonstrated *in-vitro* and in animal models. Modulation of the AMP-activated protein kinase (AMPK)-mammalian target of rapamycin (mTOR), nuclear factor of activated T cells (NFAT) or nuclear receptor (NR4A) pathways is able to block or reverse the development of detrimental epigenetic scars. Similarly, drugs that directly modify epigenetic enzymes, such as those that inhibit histone deacetylases (HDAC) inhibitors, DNA hypomethylating agents or modifiers of the Nucleosome Remodeling and DNA methylation (NuRD) complex or Polycomb Repressive Complex (PRC) have demonstrated capacity to restore host immunity in the setting of cancer-, LCMV- or murine sepsis-induced epigenetic-mediated immune suppression. A third clinically feasible strategy for reversing detrimental epigenetic scars includes bioengineering approaches to either directly reverse the detrimental epigenetic marks or to modify the epigenetic enzymes or transcription factors that induce detrimental epigenetic scars. Each of these approaches, alone or in combination, have ablated or reversed detrimental epigenetic marks in *in-vitro* or in animal models; translational studies are now required to evaluate clinical applicability.

## Introduction

Epigenetic mechanisms guide gene expression to maintain homeostasis by balancing the nature of expressed and non-expressed genes. This balance can be perturbed either by pathogen- induced epigenetic changes, such as through Rv1998 antigen secreted by *Mycobacterium tuberculosis* (*Mtb*) (1) or by chronic and severe stimulation of the immune system as in case of LCMV (2), HCV (3), sepsis (4), Schistosomiasis (5) and TB (6). Long-lasting immune suppression that follows severe or chronic infections increases the risk for secondary infections. This was recognized in 1909 when German researchers noted that TB recurrence occurred after measles (7). In the 1950s, clinicians reported an increased risk for histoplasmosis reactivation among patients recovering from TB (8). Similarly, after surviving sepsis, host immunity remains in a suppressed state that increases the risk for secondary bacterial infections and doubles mortality risk (9, 10). Survivors of pneumonia have increased risk of death with the severity of pneumonia correlated with mortality risk (11). TB survivors also have increased risk of mortality, not only from secondary infections and recurrent TB, but from increased risk of cardiovascular disease and cancer (12, 13). Although epigenetic immune suppression is needed acutely to temper exuberant immunity (14), these immunosuppressive epigenetic marks are long-lived and are thought to be a major contributing factor for increased secondary infections long after resolution of the first insult (12, 15–17). Proper epidemiological studies matched with translational studies need to be conducted, but epigenetic-mediated post-infectious myeloid and lymphoid immune suppression is a suspected explanation for why individuals retain increased mortality risks even after successful treatment for pneumonia, TB or sepsis (11, 13, 15).

After chronic and severe infections, CD4^+^ T cells are characterized as being anergic and CD8^+^ cells as being exhausted (18, 19). Functionally, anergic CD4^+^ T cells fail to recognize and respond to foreign antigen, as measured by decreased antigen-induced cellular proliferation and cytokine production (18). Similarly, CD8^+^ T cell immune exhaustion is defined by decreased antigen-induced proliferation, cytokine production and an increase in immune checkpoint inhibitors (19). Myeloid cell immune tolerance is defined by a decreased responsiveness, usually measured by decreased phagocytic capacity, killing capacity and cytokine production, e.g., TNF, IL-6 and IL-1β (10, 20–22). Animal models have demonstrated that post-infectious immune suppression is epigenetically mediated and that the detrimental epigenetic marks induced by chronic infections overlap with those induced by cancer (5, 6, 23–30). There are many studies demonstrating how cancer induced epigenetic-mediated immune suppression can be reversed. Herein, we review the growing literature of *in-vitro* and animal model studies demonstrating how to block or reverse infection induced epigenetic-mediated immune suppression and postulate how these approaches could become clinically relevant to decrease post-infectious morbidity and mortality.

## Epigenetic Mechanisms and Gene Expression

Epigenetic mechanisms are one major means of regulating gene expression. This regulation comes from nucleosomal scaffolding of the negatively charged DNA around positively charged proteins, called histones, present as two functional copies apiece of the type H2A, H2B, H3 and H4. Each nucleosome is further condensed in a higher-order structure, the chromatin. Both nucleosome and chromatin can guide accessibility of molecular factors to the DNA, thus resulting in differential gene expression (31). Cells can either circumvent or reinforce these barriers, depending on the context, by dynamically modifying DNA and histones at specific nucleotide or amino acid residues, transiently creating regions of the genome differentially accessible to gene expression machinery. Histones are modified on their free N-terminal tails, or their globular domains that physically interact with the DNA, through chemical modifications including acetylation, methylation, phosphorylation, ubiquitylation, acylation, hydroxylation, glycation, serotonylation, glycosylation, sumoylation and ADP-ribosylation (32). DNA is methylated at cytosine and adenine residues. Epigenetic marks other than acetylation and methylation are not as well studied and are less understood. The gene expression implications of certain epigenetic marks are well established. DNA methylation directly interferes with the binding of DNA and transcription factors, or it can attract proteins that bind specifically to modify DNA, thereby blocking other transcription factors from binding the site (33). Acetylation of histones H3 and H4 relaxes the nucleosome compactness and leads to partial de-condensation of chromatin locally, making the DNA more accessible. Such accessibility is referred to as “permissive” and the loss of accessibility and increased compactness referred to as “restrictive” (34). Histone modifications can be either permissive or restrictive. For example, trimethylation of histone 3 at lysine 4 (H3K4me1, H3K4me3) promotes open chromatin, while trimethylation of histone 3 at lysine 27 (H3K27me3) and at lysine 9 (H3K9me3) promotes restrictive heterochromatin (35).

## Signaling Pathways That Induce T Cell Immune Exhaustion

**Figure 1:**
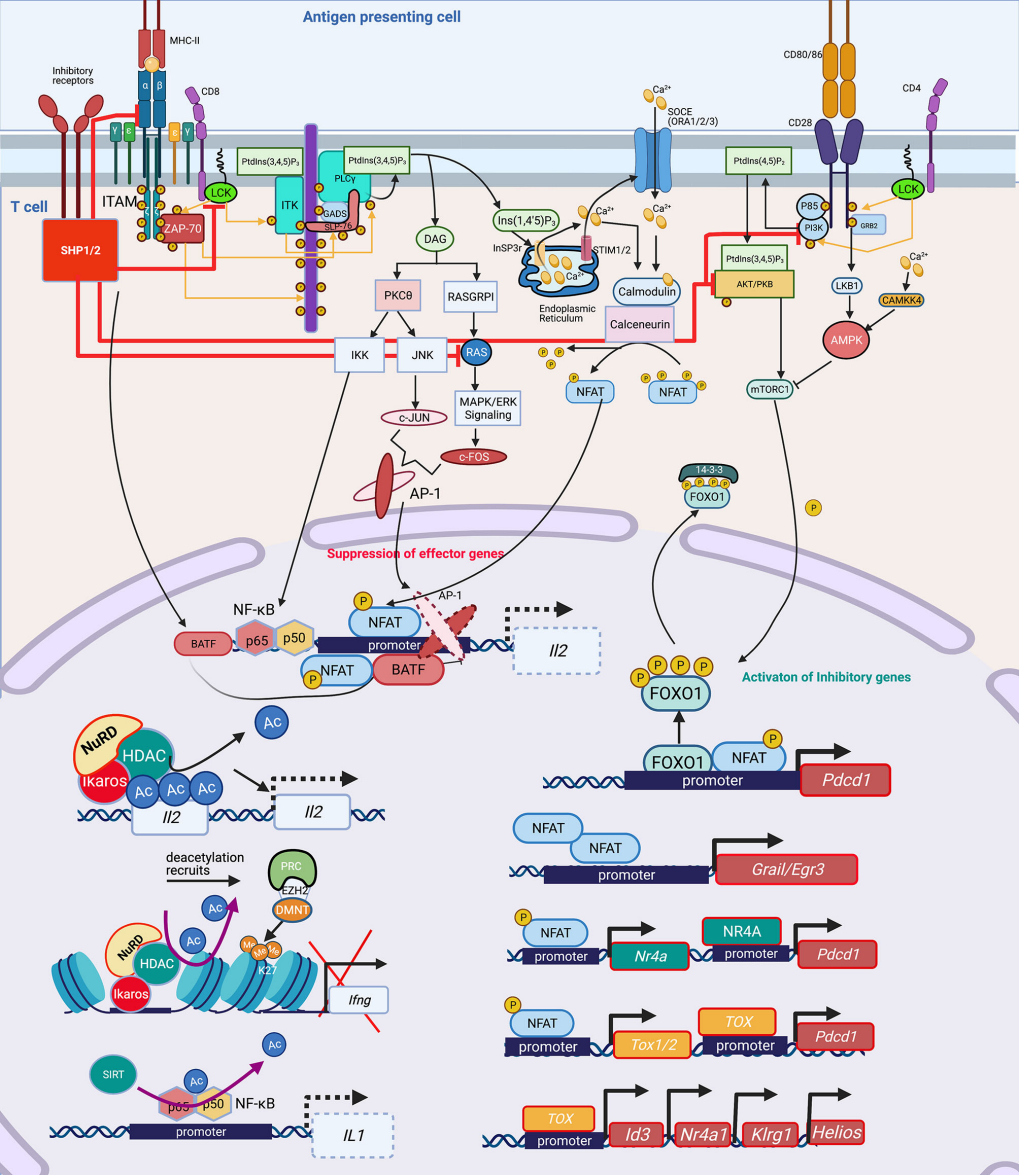
Signaling cascade and transcription factors that mediate epigenetic changes that inhibit host immunity. In T cells, protein kinase Lck and ZAP-70 initiate a signaling cascade that result in activation of PLCγ1 and production of InsP3 (IP3), a second messenger, binding to the InSP3 receptor on the ER leading to release of Ca^2+^ from the ER. The reduction of Ca^2+^ activates STIM, which recruits SOCE such as ORAI in the plasma membrane. Opening of ORAI channels in the plasma membrane results in sustained Ca^2+^ influx and activation of several Ca^2+^ regulated enzymes such as serine/threonine phosphatase calcineurin, which dephosphorylates NFAT enabling its translocation to the nucleus where it binds to promoters of effector genes including *Il2*. NFAT requires AP-1 generated through another second messenger DAG activation of PKCΦ and RAS/MAPK/ERK1 pathways. Lck also mediates activation of PI3K activating AKT and mTOR, which govern the phosphorylation of FOXO1. Phosphorylated FOXO1 is transported out of the nucleus and exists in complex with 14-3-3 in the cytoplasm. In exhausted T cells, either through activation of inhibitory receptors such as PD-1, CTL4, a dephosphorylating protein SHP1/2 is activated, which dephosphorylates Lck and ZAP70, suppressing the subsequent signaling cascades. SHP2 inhibits among others RAS, AKT, PI3K and even the TCR-MHCII microcluster, thus weakening or abrogating the effector signals at multiple levels (red inhibition arrows). This leads to widespread change in the cellular TF landscape. SHP1/2 activate BATF3, a TF, due to non-availability of AP-1 to partner with NFAT. Partnerless NFAT, alone leads to transcription of inhibitory genes and receptors including *pdcd1*, which is also transcribed by increased nuclear retention of unphosphorylated FOXO1 in the nucleus, in absence of a PI3K/AKT/mTOR activation. Unpartnered NFAT transcribes, *Nr4A* and *TOX1/2*, which further contribute to inhibitory signaling by increasing transcription of *Pdcd1*. NFAT homodimer transcribes inhibitory genes *Grail3/Erg3*. TOX leads to transcription of genes such as *Id3*, *Nr4a1*, *Klrg1*, *Helios*. Many of these genes and TF lead to epigenetic modifications, which further contribute to exhausted phenotype. TF, Ikaros (Helios family) can directly bind to the *Il2* promoter and recruit NuRD, which has HDAC and deacetylates *Il2* leading to its transcriptional repression. Deacetylation is usually followed by recruitment of PRC, which through EZH2 can further add to closing of chromatin by adding methylation marks at H3K27, as seen at the *Ifng* locus. Another, NAD: NADH+ dependent deacetylase, SIRT can directly deacetylate NF-κB to decreases *IL1* transcription. Lck, LCK proto-oncogene, Src family tyrosine kinase; ZAP-70, zeta chain of T cell receptor associated protein kinase 70; PLC, Phospholipase C; IP3/InsP_3_, inositol 1,4,5-trisphosphate; ER, Endoplasmic reticulum; STIM, stromal interaction molecule 1; SOCE, Store-operated calcium entry; ORAI, ORAI calcium release-activated calcium modulator; NFAT, Nuclear factor of activated T-cells; IL, Interleukin; AP-1, Activator protein1; DAG, Di-Acyl Glycerol; PKCΦ, Protein kinase C; MAPK, Mitogen-Activated Protein Kinase; FOXO1, Forkhead box protein O1; 14-3-3, tyrosine 3-monooxygenase/tryptophan 5-monooxygenase activation protein theta (encoded by *YWHAQ*); PD-1/Pdcd, programmed cell death 1; CTL4, Cytotoxic T-Lymphocyte Associated Protein 4; SHP, Src homology 2 domain-containing tyrosine phosphatase 2; PI3K, phosphatidylinositol 3-kinase; AKT, Protein kinase B; mTOR, mammalian target of rapamycin; MHC, Major Histocompatibility complex; TF, Transcription factors; BATF, Basic Leucine Zipper ATF-Like Transcription Factor; Nr4A, Nuclear Receptor Subfamily 4 Group A Member 1; TOX, Thymocyte Selection Associated High Mobility Group Box; Erg, ETS transcription factor ERG; Id3, Inhibitor Of DNA Binding 3; Klrg1, Killer Cell Lectin Like Receptor G1; NuRD, Nucleosome and DNA Remodeling complex; PRC, Polycomb Repressive Complex; EZH2, Enhancer Of Zeste 2 Polycomb Repressive Complex 2 Subunit; H3K27me3, H3 lysine 27 trimethylation; Ac, Acetylation; Me, Methylation. Created with BioRender.com.

Broadly, T-cell activation involves tightly controlled signaling pathways and cascades, that when perturbed lead to transcription factor (TF) imbalances that then drive epigenetic-mediated gene expression inhibition. Activation of the T-cell receptor (TCR) by MHC-antigen complexes assembles the “TCR signalosome” that results in downstream phosphorylation events and activation of secondary signaling molecules (18, 36–40). Downstream of the TCR, key events include phosphorylation of tyrosine kinases and phospholipase C (PLC)1 (41–43). Activated PLCγ1 cleaves phosphatidylinositol 4,5-bisphosphate (PIP_2_) to diacylglycerol (DAG) and inositol 1,4,5-triphosphate (IP3), the former being critical for proper activation of activator protein-1 (AP-1) complex, a heterodimer of c-Fos and c-Jun (**Figure 1**) (44–47). IP3 plays a central role in calcium signaling by releasing intracellular calcium stores from the endoplasmic reticulum (ER) (48) and thereby dephosphorylating NFAT proteins which translocate to the nucleus, and bind with their transcriptional partners, such as AP-1, to activate distinct ranscriptional programs (**Figure 1**) (49). NFAT/AP-1 transcriptional complexes bind to the promoters of various cytokine genes, including *Il2*, leading to their active transcription. In the absence of co-stimulation, impaired AP-1 activation results in NFAT homodimerization inducing transcriptional and epigenetic changes that yield anergic and exhausted T cells (**Figure 1**) (18, 44, 50, 51).

Activation of the TCR and CD28 co-stimulatory induces a flux of intracellular Ca^2+^ and activation of the PI3K-AMPK-mTOR signaling pathway. Activated mTOR engages several downstream effector pathways, including promoting metabolism by activating gene expression of the TFs hypoxia-inducible factor 1α (HIF1α), MYC and sterol regulatory element-binding protein (SREBP). Upregulation of inhibitory receptor signaling recruits Src homology 2 domain-containing tyrosine phosphatase 2 (SHP2) phosphatase which interferes with CD28 costimulatory signaling by blocking PKC-θ (52) and PLCγ1 (53). Increased programmed death protein 1 (PD-1) inhibits AKT and mTOR pathways (54), activates Basic leucine transcription factor (BATF) to repress T-cell proliferation and cytokine secretion in HIV-specific CD8+ T cells (55), and inhibits IL-2 production to limit T-cell proliferation (52). NFAT1 in the absence of AP-1 interaction promotes the expression of *Pdcd1* (PD-1 encoding gene) (56). Inactivation of the AKT/mTOR pathway promotes FOXO1 retention in the nucleus to enable continued inhibitory receptor *Pdcd1* gene transactivation (57). PD-1 signaling through SHP2 activates AMPK, which is an inhibitor of mTOR signaling (already abrogated by inactivation of PI3K and AKT), leading to downregulation of HIF-1α and MYC, which in turn governs the transcription of the glycolytic enzymes such as GLUT1, thereby decreasing cellular metabolism.

## Transcription Factors Driving T Cell Immune Exhaustion

NFAT homodimers play a critical role in induction of the T cell anergy transcriptional program (44). NFAT1 homodimers bind to specific NFAT binding sites on T cell anergy-associated gene promoters. For example, NFAT homodimer consensus binding sites are present in the promoter of *Grail*, a T cell anergy-associated gene (50). Expression of the early growth response gene 2 (Egr2) and Egr3 is NFAT-dependent, and these TFs are associated with regulation of gene expression of the Casitas B-lineage lymphoma b (Cbl-b) E3 ubiquitin ligase in anergic T cells (58). Downstream of NFAT signaling, the TF Ikaros, binds to the *IL2* gene locus, and recruits the NuRD complex, including histone deacetylases (HDACs), thereby facilitating epigenetic remodeling, specifically histone deacetylation, of the *Il2* promoter, thus effectively silencing gene expression (59, 60).

Highlighting the critical importance of a balanced NFAT response, a bioengineered constitutively active form of NFAT, termed CA-RIT-NFAT1, closes chromatin conformation inducing epigenetic-mediated immune exhaustion, including decreased microbial killing capacity in CD8^+^ T cells (56, 61). Constitutively active NFAT1 leads to the enrichment of genes belonging to the nuclear receptor (NR) family of genes, specifically members of the NR4A family. In particular, NR4A2 (NURR1) and NR4A3 (NOR1) exhibit high enrichment upon CA-RIT-NFAT1 expression. NR4A family member genes exhibit greater chromatin accessibility in exhausted tumor infiltrating lymphocytes (62). Using a chimeric antigen receptor T cell (CAR T cell) model, a NR4A triple knockout reversed detrimental chromatin accessibility, and promoted tumor regression and prolonged survival of tumor-bearing mice, thus illustrating the epigenetic and functional relevance of the NR4A family in T cell exhaustion. NR4A is also important for PD-1 and TIM3 expression, markers of T cell exhaustion (62).

NFAT homodimers also induce the thymocyte selection-associated high mobility group box (TOX) proteins which mediate the expression of inhibitory receptors such as PD-1 and TIM3, leading to the T cell exhaustion phenotype. Increased TOX expression occurs in chronic infection models such as LCMV and chronic hepatitis C (HCV) infection (51, 63). Removal of the nuclear localization sequence (NLS) and part of the DNA-binding domain from TOX *via* deletion of exon 5 resulted in decreased PD-1 expression and impaired generation of the T cell exhaustion phenotype. In addition, TOX exon 5 deletion resulted in differential expression of genes associated with T cell exhaustion such as *Id3, Helios (Ikzf2), Nr4a1, Nr4a2, Pdcd1*, and *Klrg1*. Conversely, over-expressing TOX in healthy T cells increases PD-1 expression, demonstrating a role in inducing the T cell exhaustion phenotype (63). Deletion of TOX exon 5 leads to decreased chromatin accessibility of the *Pdcd1* gene locus, which encodes PD-1, and increased chromatin accessibility to *Tnf* (63). Knocking out *Tox* in CD8^+^ CAR tumor infiltrating lymphocytes (TILs) increase cytolytic activity further supporting the notion that TOX specifically attenuates CD8^+^ T cell effector function (**Figure 1**).

These studies provide evidence for the importance of the NFAT, TOX and NR4A TFs in driving epigenetic-mediated immune exhaustion, and also suggest strategies to alter their activation could be therapeutically pivotal (**Figure 1**). For example, Cyclosporin A (CsA), a calcineurin inhibitor, inhibits NFAT activation, thereby inhibiting TOX, NR4A1, NR4A2 and NR4A3 and the subsequent detrimental chromatin conformation changes that leads to immune exhaustion (51). Tacrolimus (binding to FK506) inhibits calcineurin by a different mechanism, but similarly decreases NFAT and TOX, blocking the chromatin confirmation changes that upregulate PD-1 and LAG3, thereby preserving capacity to produce TNF and IFN (64–66). Discussed in more detail below, bioengineered upregulation of c-Jun rescues NFAT-AP-1 imbalance, thereby restoring immune function. Therefore, while tacrolimus and CsA are considered immune suppressants, they can prevent detrimental chromatin conformation that leads to immune exhaustion, thereby preserving host immunity. Studies are needed to evaluate if these agents could be of benefit in humans following pneumonia, sepsis or TB.

## Signaling Pathways That Induce Myeloid Immune Tolerance

**Figure 2:**
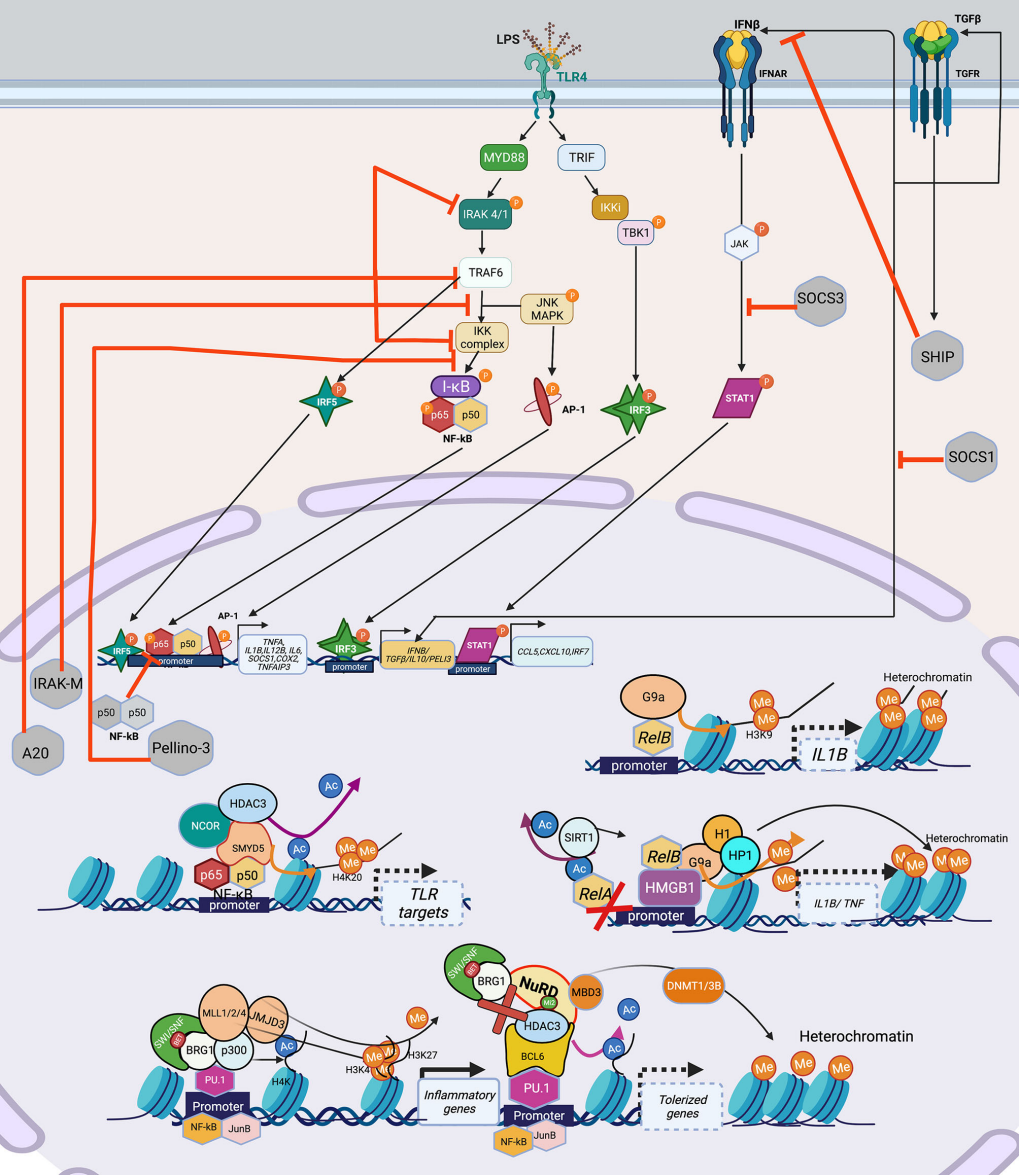
Signaling cascade and transcription factors that mediate epigenetic changes that inhibit host immunity in myeloid cells. TLR4 recognizes LPS, and engages the MyD88-TRIF pathway to induce the TFs: NF-κB, AP-1, IRF3, IRF5 which leads to the induction of pro-inflammatory genes such as *TNFA, IL1B, IL6, COX2* etc. IRF3 induces the production of IFNβ and TGFβ, which adds to the IFN signaling and induces STAT1 leading to transcription of *CCL5, CXCL10 and IRF7*. Overwhelming LPS stimulation as seen in sepsis, leads to lesser production and engagement of TLR4 and its pathway components, with over-inflammation leading to production of inhibitory molecules such as IRAK-M, A20, Pellino-3, SHIP, which inhibit various parts of the LPS-TLR4 signaling cascade, leading to a tolerized phenotype. Epigenetically, multiple mechanisms have been shown to lead to and maintenance of the tolerized phenotype. Guided by TFs such as NF-κB and its isoform RelB, which can recruit HDACs (SIRT included) either alone or in a repressome complex, usually with a chromatin modifier such as SWI/SNF results in deacetylation of histones, followed by addition of repressive methylation (H3K9, H3K3) by DNMT such as SMYD5 (in the NCOR-HDAC3 repressome), or KMT such as G9a bound to HMGB1 (can recruit H1 and HP1) to close the chromatin and suppress gene expression. Lineage TFs such as PU.1 provide good example of this assembly of the SWI/SNF complex containing BRG1 which can recruit HAT (p300) to acetylate H4K, HMT (MLL1/2/3) to add permissive H3K4 and demethylase such as JMJD3 to remove repressive H3K27 to activate inflammatory genes upon LPS stimulation. The same PU.1 when bound to co-repressor BCL6 can induce tolerance by losing the SWI/SNF complex and recruitment of NuRD, which recruits HDAC3 to remove acetylation and induce *de novo* methylation *via* DNMT1/3B to close the chromatin and thus shutting down inflammatory gene transcription in tolerance. Created with BioRender.com. TLR4, Toll-like receptor 4; LPS, Bacterial Lipopolysaccharide; MyD88, myeloid differentiation factor 88; TRIF, TIR-domain containing adapter-inducing interferon; TF, Transcription factor; STAT1, signal transducer and activator of transcription; IRAK, interleukin-1 receptor-associated kinase; TRAF6, TNF Receptor Associated Factor 6; SHIP; SH2 domain-containing inositol phosphatase 1; IKK, Ikappa B Kinase; TBK, TANK-binding kinase 1; MAPK, Mitogen-Activated Protein Kinase; I-KB, nuclear factor of kappa light polypeptide gene enhancer in B-cells inhibitor; IRF, Interferon regulatory factors; SWI/SNF, SWItch/Sucrose Non-Fermentable; BRG1, Brahma-related *gene*-1; HDAC, Histone deacetylase; H1, *H1*.1 Linker Histone; HP1,Heterochromatin protein-1; HMGB1, High Mobility Group Box 1; DNMT, DNA methyltransferase; MLL, mixed lineage leukemia (lysine methyl transferase); JMJD, Jumonji domain containing protein; BCL6, B-cell lymphoma 6; MBD3, Methyl-CpG Binding Domain Protein 3.

For myeloid cells, the best studied model for immune tolerance is LPS challenge or sepsis. The exact myeloid tolerance mechanism(s) has not yet been elucidated for other infections. Thus, we discuss LPS/sepsis as a central theme for the signaling pathways inducing myeloid tolerance. Following overstimulation, myeloid cells, including monocytes and macrophages, develop tolerance, a state of cell refractoriness defined by an inability to mount an inflammatory response to a secondary stimulation (67). Pathogen- or danger-associated molecular patterns (PAMPs/DAMPs) such as LPS are sensed *via* pattern recognition receptors (PRR), e.g., toll-like receptors (TLR). LPS is recognized by TLR4, mediating signaling through two distinct adaptor pathways, myeloid differentiation factor 88 (MyD88) and TIR-domain containing adapter-inducing interferon (TRIF). The MyD88 pathway employs interleukin-1 receptor-associated kinase (IRAK)1 and 4 kinases and TNF receptor-associated factor (TRAF) 6 to activate NFκB and AP-1 signaling, promoting transcription of pro-inflammatory cytokines. Activation of TRIF pathway leads to janus kinase (JAK)/signal transducer and activator of transcription (STAT)1 and type I interferon activation and increases the expression of interferon-inducible genes such as *TNFA, IFNB, IL1B, IL6*, and *COX2* (68, 69). PI3K interacts with MYD88 and also influences TLR4 signaling (70). LPS-induced myeloid tolerance involves downregulation of TLR4 expression, decreased recruitment of MyD88 or TRIF to TLR4, decreased activation of IRAK1/4 and diminished canonical NFκB signaling (p65/p50 heterodimer) *via* formation of inactive p50 homodimers (67, 71), decreased AP-1, reduced expression of *TNFA, IL1B, IL6 and IL12B*, and increased expression of *IL10* and *TGFB1* (**Figure 2**).

LPS-tolerized myeloid cells are also characterized by negative regulatory molecules IRAK-M, A20, SH2 domain-containing inositol phosphatase 1 (SHIP1) (72), Pellino-3 (73), suppression of tumorigenicity 2 (ST2) (74), suppression of cytokine signaling (SOCS)1 and SOCS3 that inhibit TLR signaling (67, 69) (**Figure 2**). PI3K pathway, activated in LPS tolerance, also contributes to production of anti-inflammatory cytokines such as sIL-1RA (75) and its inhibition with wortmannin mitigates tolerance and increases TNF production (76). NFκB upregulates HDACs that remove histone acetyl marks and recruit the NuRD complex with the net result of a “repressome” such that euchromatin marks (e.g., histone acetylation) are removed and heterochromatin marks (e.g., DNA methylation and H3K9 and H3K27me) are induced (**Figure 2**) (20, 77–79). MyD88 activation, *via* non-coding RNAs, also contributes to decreased chromatin accessibility changes thereby inducing tolerance (80, 81). Acutely, tolerance is beneficial as studies have demonstrated that inhibiting post-sepsis epigenetic-mediated immune suppression too early exacerbates immune pathology (14).

## Transcription Factors Driving Myeloid Immune Tolerance

Similar to the situation with immune exhaustion, myeloid cell immune tolerance is mediated by TFs that recruit co-activator and corepressor complexes that modify chromatin accessibility through post-translational modifications (**Figure 2**). The myeloid lineage defining transcription factor PU.1 facilitates chromatin opening with an increase in H3K4me3 at promoters and H3K4me1 at enhancers. However, in resting macrophages, corepressors such as B-cell lymphoma 6 (BCL-6) associate with PU.1 and recruit HDACs and histone demethylase resulting in repression of many LPS-inducible genes (82). In an analysis of LPS-induced tolerant and non-tolerant genes, NF-κB and MAPK were downregulated in tolerant macrophages (83). NFκB family TF isoform, RelB, mediates epigenetic silencing *via* facilitating the direct deposition of repressive histone marks by the H3 lysine methyltransferase (KMT) G9a at the *IL1B* promoter (78). Similarly, binding of high-mobility group box-1 protein (HMGB1) and histone H1 linker at the promoters of *TNF* and *IL1B* genes leads to transcription silencing by promoting assembly of RelB, which results in deposition of H3K9me2 mediated by the KMT G9a. Depletion of HMGB1 by siRNA results in dissociation of RelB from the promoter and partially restores *TNF* transcription (84).

Tolerized myeloid cells exhibit decreased chromatin accessibility due to decreased TLR-induced recruitment of the BRG1-containing SWI/SNF nucleosome remodeling complex and changes in histone acetylation and methylation (83). The NCoR-Hdac3-p50 repressome contains histone deacetylase and SET histone methyltransferases (SMYD5) that result in H3K9/14 deacetylation (77) and H4K20 methylation (85) respectively, both contributing to heterochromatin and repression of tolerizeable genes, thereby inhibiting the expression of genes downstream of TLR4 activation. Genetic disruption of the NcoR-Hdac3 interaction abolishes TLR4 tolerance (83). Interestingly, IFN-γ prevents tolerance by preserving expression of the receptor-interacting protein 140 (RIP140) coactivator and promoting TLR-induced chromatin accessibility upon secondary TLR challenge (86). In contrast, non-tolerized genes maintain an open chromatin state and exhibit more H4 acetylation and maintain H3K4me3 after re-stimulation (83). Interestingly, the NuRD complex acts antagonistically, and in a SWI/SNF-BRG1 dependent manner in LPS stimulated macrophages showing that these complexes exhibit concerted action to guide gene expression in myeloid cells (87) (**Figure 2**).

In summary, PU.1 facilitates myeloid gene transcription, while tolerance is associated with binding of co-repressor BCL-6 to PU.1, disruption of the NFκB active heterodimer and epigenetic silencing *via* HMGB1, RelB, NCoR-HDAC3-p50 repressome (**Figure 2**), increased SMYD5 and G9a methyltransferase and decreased chromatin accessibility due to reduced recruitment of BRG1-NRC. BET inhibitors (that bind to the bromodomain in the BRG1-NRC) such as IBET151, rescue tolerance in a preventative way when administered along with LPS, and not post LPS exposure (88).

Thus, HDAC inhibitors and G9a inhibitors if given after the resolution of acute infection, could potentially mitigate aspects of long-lived myeloid cell tolerance, while BET inhibitors act in a more preventative way (88).

## Metabolic Mechanisms Leading to Immune Exhaustion and Tolerance

**Figure 3:**
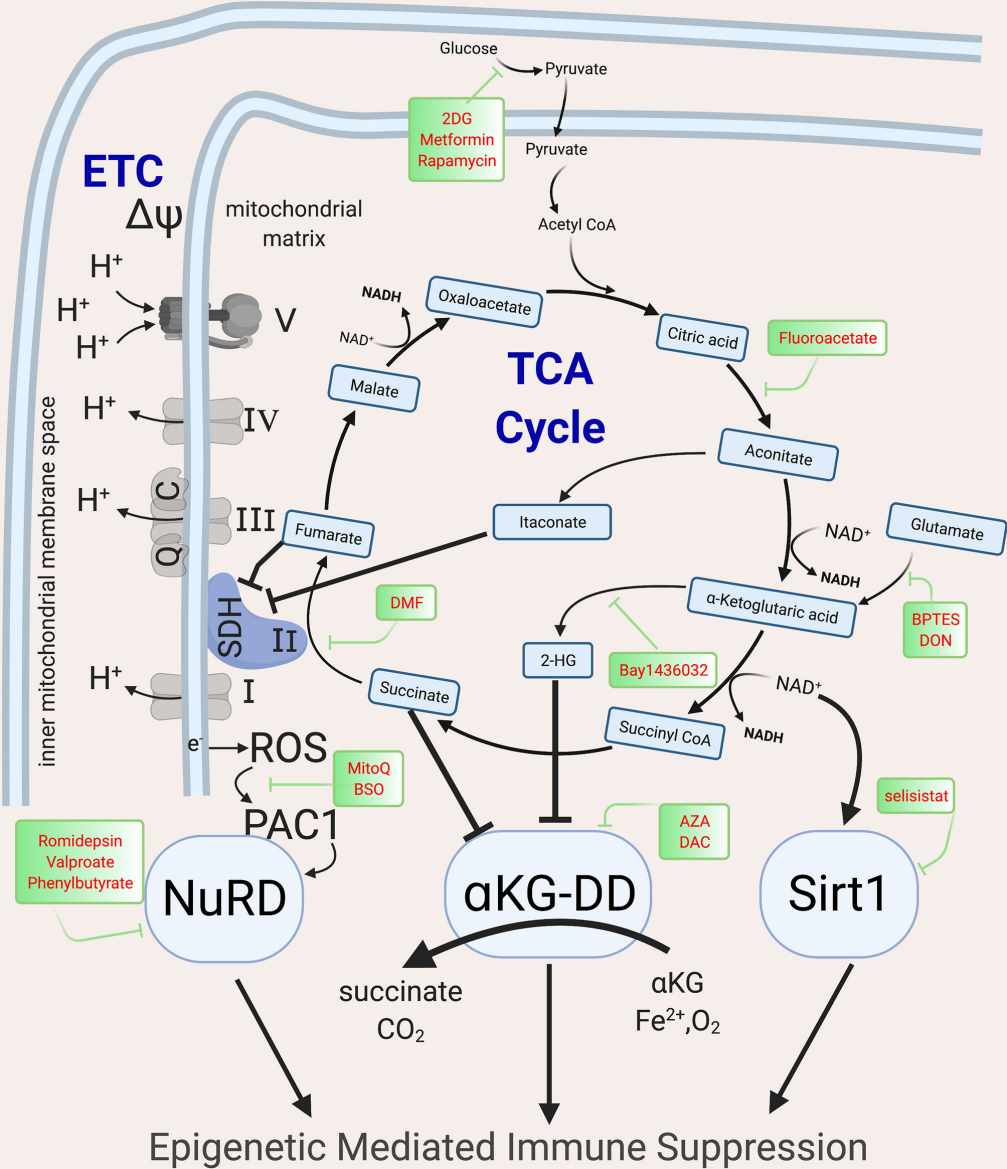
Metabolic intermediates of the TCA cycle guide epigenetic changes that inhibit host immunity. The TCA cycle metabolites act as co-factor for major epigenetic enzymes that shape the epigenomic landscape post infection *via* three overlapping and redundant major metabolic-epigenetic rheostats (RST). RST1: NAD+: NADH-SIRT: Dependent on the level of NAD+ in the cell, Sirtuins, which are histone deacetylases, can remove acetyl groups and lead to immune suppression; RST2: Succinate-αKG-αKGDD: Dependent on the levels of α−ketoglutarate and succinate (which along with fumarate, malate and Itaconate acts as inhibitors of KGDDs), leads to the activation/inhibition of a family of enzyme dioxygenases which regulate the DNA methylation levels by methylating (via DNMTs) and demethylate (via KDM, JMJD and TET) the DNA; RST3: ROS-PAC1-NuRD: guided by the activation of the ETC, which leads to electron leak and induction of ROS and activation of NuRD, which is multiprotein complex guiding DNA methylation and chromatin accessibility. The main enzymes of the TCA cycle and the ETC are shown, along with the drugs that can be used to block specific enzymes and help with reversing epigenetic mediated Immune suppression. NAD, Nicotinamide adenine dinucleotide; SIRT, Sirtuins; αKGDD, α−ketoglutarate-dependent-dioxygenases; DNMT, DNA methyl transferase; KDM, Histone demethylase; JMJD, Jumonji domain containing protein; ROS, reactive oxygen species; PAC1, Phosphatase of activated cells 1; NuRD, Nucleosome Remodeling and DNA methylation complex; TCA, Tri-carboxylic acid cycle; ETC, Electron transport chain. Created with BioRender.com.

Upon immune activation *via* mTOR and NFAT signaling, shifts in cellular metabolism increase glycolysis, the tricarboxylic acid cycle (TCA, also known as the Krebs cycle) and electron transport chain (ETC), not only to meet high energy demands for proliferation and effector function, but also to produce the intermediate metabolites that fuel the biosynthesis of effector protein functions (10, 89–91). While initially beneficial, in severe or chronic infection, these metabolic shifts contribute to epigenetic changes that induce immune suppression (35, 89, 90, 92–95). In both lymphoid and myeloid cells, these metabolic shifts are mediated by the PI3K-Akt-mTOR pathway (96) and if the infection persists, the associated signaling cascades are downregulated and epigenetic mechanisms suppress host immunity, with loss of accessible chromatin that allows for expression of cytokines such as *Tnf* and *Ifng* and gain of chromatin accessible regions in inhibitory loci such as *Pdcd1* (97, 98), thereby placing cells into an immune suppressed state (97). The described metabolic shifts induce epigenetic changes due to alteration in metabolic precursors required for epigenetic marks. At least three overlapping metabolic-epigenetic rheostats (**Figure 3**) have been identified that regulate host immunity (99–101).

Histone acetylation, an epigenetic mark characteristic of euchromatin is regulated by the availability of nicotinamide adenine dinucleotide (NAD^+^). High NAD^+^ levels and NAD^+^/NADH ratios induce the NAD-dependent deacetylase sirtuins (SIRTs) which deacetylate both histone and non-histone proteins. High dose LPS exposure, *via* upregulation of IDO1-induced *de novo* synthesis of NAD^+^, activates SIRT1, leading to histone deacetylation and gene silencing of proinflammatory genes such as *Tnf* and *Il1b* (102, 103). SIRT1 deacetylates p65 lysine of RelA (NF-κB) and nucleosomal H4K16 to terminate NF-κB dependent transcription and remains bound to assembled RelB and recruited transcriptional repressor complex (including heterochromatin linker H1) generating tolerance (102). NAD levels are regulated by CD38 and IDO1. CD38 levels correlate with T cell exhaustion (104, 105) and are elevated in patients with Cytomegalovirus (CMV) (106), Epstein-Barr-virus (EBV) (107), mycobacteria (108) and HIV, and are associated with poor prognosis (109). CD38, an extracellular and intracellular NADase, converts NAD^+^ molecules to a single cyclic ADP ribose thereby drastically shifting NAD^+^/NADH ratios, and activating sirtuin-mediated epigenetic mediated immune suppression (104). IDO, another mediator of immune suppression, is the rate limiting enzyme step in *de novo* NAD synthesis, converting tryptophan to kyneurine. IDO, elevated in sepsis and TB, inhibits host immunity by decreasing nuclear NAD^+^ concentrations, and initiating sirtuin activation (103, 110). Sirtuins regulate post-infectious immune suppression in both lymphoid and myeloid cells (102, 111), with inhibitors of CD38, IDO1 or SIRT1 able to restore host immunity and prevent mortality in animal models (104, 110, 112).

The second metabolic-epigenetic immune rheostat mechanism is guided by the balance of α-ketoglutarate (α-KG) and succinate. DNA methyltransferases (DNMT), lysine demethylase (KDM), jumonji domain-containing protein D3 (JMJD3) and Ten-eleven translocase (TET), require α-KG (also known as known as 2-oxoglutarate-2OG) as a co-substrate (113). Therefore, these epigenetic enzymes are known as α-KG or 2OG dependent dioxygenases (α-KG-DD) (113). Succinate, the end product of these chemical reactions, acts as a negative feedback loop to inhibit their function (114). In addition to succinate, other late-stage TCA metabolites including fumarate, malate, itaconate and 2-hydroxyglutarate (2HG) inhibit the α-KG-DD epigenetic enzymes (115–120). The importance of TCA metabolites in epigenetic regulation was first described in cancer where mutations in IDH, succinate dehydrogenase (SDH) or fumarate hydratase were found to induce global epigenetic disturbances (121, 122). These mutations lead to TCA metabolite imbalances that drive global DNA and histone hyper-methylation and immune tolerance (123–126). While originally described in cancer, studies in wild type mouse and human healthy T cells demonstrate that, upon immune activation, 2HG is increased *via* Von Hippel-Lindau (VHL)-HIF1α (120). Initially, 2HG increases T cell IL-2 production, but when 2HG elevations persist, there are global increases in the inhibitory epigenetic mark H3K27me3 with suppression of T cell cytotoxic function (120). Dimethyl fumarate (DMF), an immune suppressive therapy for multiple sclerosis, induces DNA methylation and heterochromatin in monocytes and T cells to suppress exuberant immunity (127, 128).The timing and duration of TCA metabolite shifts need to be further explored as short term shifts induce immune beneficial immunity, while others are immune suppressive (118, 120, 129, 130).

The known metabolic-epigenetic immune rheostats are overlapping and redundant, as demonstrated by the third known metabolic-epigenetic rheostat. SDH is unique in that it is both part of the TCA cycle and the ETC. Upon immune activation, the increase in glycolysis fuels the ETC and when persistent, electrons leak out of the inner mitochondrial space (**Figure 3**), increasing reactive oxygen species (ROS) in the mitochondrial matrix. This increase in ROS triggers phosphatase of activated cells 1 (PAC1, encoded by *DUSP2*, dual specificity protein phosphatase 2) and nuclear factor erythroid 2-related factor (NRF2, encoded by *NFE2L2*) to activate the NuRD complex (131, 132). The NuRD induces histone deacetylation and DNA hypermethylation (*via* MBD2/3) and is instrumental in limiting exuberant immune pathology in macrophages after sepsis and preventing T cell autoimmunity (133). Mice with tumor-induced immune exhaustion demonstrate elevated mitochondrial ROS that correlates with detrimental epigenetic marks (DNA hypermethylation and closed chromatin conformation) and immune suppression (134). Inhibiting mitochondrial ROS accumulation ablates immune suppression (92, 135), however to date, the direct link *via* the NuRD has not been demonstrated.

The immune inhibitory effects due to itaconate further demonstrate the overlap of mechanisms by which metabolism acts as an immune rheostat. The direct epigenetic effect of itaconate has not yet been described. Itaconate is produced by diverting cis-aconitate in the TCA cycle by the enzyme cis-aconitate decarboxylase. Itaconate inhibits SDH, leading to succinate accumulation (**Figure 3**). Therefore, it is presumed but not proven that itaconate induces epigenetic changes akin to succinate. Itaconate acts as a negative feedback to limit exuberant immune pathology, inducing NRF2 nuclear translocation and downregulation of IL1β and IL-6 (93). Upon *Mtb* infection, mice with knockdown of *Irg1* (gene that codes for CAD, the enzyme that converts citrate to itaconate) have increased proinflammatory cytokine production (IL1β, IL-6, IFN-y, IL12) and fatal exuberant pulmonary infiltration of innate immune cells (94, 95).

As noted earlier, most epigenetic marks depend upon metabolic precursors which, when altered, influence the epigenetic landscape. For example, S-adenosylmethionine (SAM)-mediated one-carbon metabolism supplies methyl groups for histone and DNMT. Chronic antigenic stimulation, as occurs in chronic LCMV infection, induces cellular metabolism from glycolysis and glutaminolysis, decreasing amino acid metabolic pathways feeding into the one-carbon metabolism such that reduced threonine, reduces SAM, leading to decreased H3K4me3 levels and impaired cytokine production (136, 137). SAM supplementation increased H4 arginine 31 methylation of STAT1 by Arginine methyl transferase (PRMT1), which is inhibited by HBV and is functionally essential for STAT1 function, improved antiviral effects of IFN-α in HBV infection (138) and HCV (139).

Current knowledge of the metabolic-epigenetic immune rheostat axes indicates functional roles in inhibiting acute exuberant immune pathology at the cost of long-lasting epigenetic marks and long-lasting immune suppression. To date, mechanistic studies have identified inhibitors of glycolysis, glutaminolysis and mTOR as well as transient glucose (140) restriction as possible means to block metabolic-epigenetic immune suppression. However, studies are needed to evaluate the clinical applicability of these mechanisms in severe and chronic infections.

## Epigenetic Drugs to Restore Immune Response

Epigenetic drugs have been developed predominantly for cancer therapeutics, however some, such as valproic acid and hydralazine are routinely used as antiepileptics and antihypertensives. Currently approved or in-development epigenetic modifying drugs include DNA hypomethylating agents (HMAs), HDAC inhibitors, lysine methyltransferase inhibitors (targeting EZH2, G9a, DOTL), and BET bromodomain (BRD) inhibitors. Several studies, most in cancer but a growing number in infectious diseases, have demonstrated that epigenetic drugs can reverse epigenetic-mediated immune suppression. Animal models, especially those for sepsis and chronic LCMV, have documented the mechanisms by which epigenetic drugs are able to restore host immunity.

Infection with clone 13 LCMV, the prototypical model for inducing CD8^+^ T cell immune exhaustion induces global DNA methylation changes associated with immune exhaustion (2, 25, 141). Applying either a conditional knock of *DNMT3a* or the hypomethylating drug decitabine was able to restore CD8^+^ T cell effector function (25). Humans with sepsis upregulate DNMT1, DNMT3a and DNMT3b, resulting in global DNA methylation differences, 82.6% of which are suppressive hypermethylated marks (142). In a cecal ligation model of murine sepsis, decitabine restored immune function and decreased mortality (142). Similarly, application of the hypomethylating drug azacytidine or decitabine to cancer cell lines increased interferon responsiveness and antigen presentation (143–145).

DNA methylation changes occur in parallel to other epigenetic modifications to inhibit host immunity. Co-immunoprecipitation studies demonstrated that DNMT associates with EZH2, the catalytic unit of the PRC (146). EZH2 methylates H3K27, inhibiting gene expression (146) and the combination of EZH2 inhibition (RNAi EZH2) and azacytidine can restore gene expression (146). EZH2 acts as an anchor point for multiple epigenetic mechanisms to suppress gene expression (**Table 1**). In ovarian cancer cell lines, inhibition of EZH2 using DZNep (inhibitor of SAM dependent enzymes), EPZ6438 and GSK126 (selective inhibitor of EZH2) and DNMT (azacytadine) synergistically increase IFN-γ responsiveness, and CXCL9 and CXCL10 expression, while shrinking tumor size (165). In melanoma, prostate cancer, hepatocellular cancer and colon cancer, EZH2-induced epigenetic marks inhibit Th1 polarization and IFN-γ-JAK-STAT signaling, with EZH2 knockdown or pharmacologic inhibition using DZNep or GSK126, restoring IFN-γ-induced gene expression (157–160). *In-vitro*, EZH2 regulates both Th1 and Th2 polarization, and inhibiting EZH2 genetically or by using EZH2 inhibitor DZNep, results in reduction of the suppressive histone mark H3K27me3, thereby augmenting both Th1 and Th2 polarization and effector cytokine production (166). In exhausted CD8 T cells, GSK126, a specific EZH2 inhibitor, restores CD8 cell cytotoxicity (164). In a CLP model of sepsis, H3K27me3, the repressive epigenetic mark induced by EZH2, persists to inhibit IL12 immunity at least 6 weeks after the initial septic insult (155). In clinical studies of sepsis, EZH2 expression increases proportional to disease severity and correlates with poor clinical outcomes (156). In TB, EZH2 is expressed early (161), with EZH2 inhibition decreasing *Ifng* and *Tnf* H3K27me2 and increasing TNF and IFN-γ production (162).

**Table 1:** Drugs targeting epigenetic enzymes restore Immunity and Reverse Epigenetic-mediated Immune suppression.

Epigenetic post translational modification	Target enzyme/Action	Drugs	Drug Mechanisms of Action	Evidence for Causing disease	Evidence for improving Infection outcome	Evidence for improving outcome Cancer
***Histone Acetylation***	Histone Deacetylases (HDAC)	Valproic acid; Sodium or phenyl butyrate; Trichostatin-A (TSA)	Reduces histone deacetylation.	Histone deacetylation limits acute immunity during chronic viral infection (147). *Mtb* suppress critical immune genes, such as IL12, by upregulating HDAC1, leading to deacetylation of histone H3 (148).	Valproic acid, restores CD8 T cell effector function and listeria killing capacity in LCMV (147). TSA and sodium butyrate restore host immunity, cytokine production restoring *Mtb* killing capacity (148). Sodium or phenyl butyrate, restores IFN-γ downstream responsiveness (149, 150), as well as inflammasome and IL1 pathway gene expression (151).	Entinostat Increases host anti-tumor immunity (152).
	(HDAC1, 3)	Entinostat (MS-275)	Entinostat, preferentially reduces histone deacetylation HDAC1 and HDAC3.			
	Sirtuins (SIRT), NAD+ dependent deacetylators of proteins, including histones	EX-527	Elective Sirt1 inhibitor prevents histone deacetylation	LPS induced immune tolerance characterized by deacetylation and silencing of TNF, IL1β and NFκB by Sirt in a NAD dependent manner (20, 102). In T cells, *Mtb*-induced upregulation of SIRT2 deacetylates NFκB to suppress immunity and *Mtb* killing capacity in mouse models (153). Increased SIRT1 confers chemoresistance (154).	When used in the acute phase of sepsis, it increases morbidity. When used in the immune hyporesponsive phase of sepsis, it is able to reduce post-sepsis mortality (112). SIRT2 inhibition improves both myeloid and lymphoid immunity and *Mtb* killing capacity in mouse models (153).	EX-527, increases chemosensitivity in cancer (154).
***DNA Methylation***	DNA methyl Transferase (DNMT)3A	Decitabine; Azacytidine	Demethylation/Hypomethylation	Upregulated *DNMT1*, *DNMT3a* and *DNMT3b*, resulting in global DNA hypermethylation methylation in sepsis (142).	Knock out *DNMT3a*, or Decabitine Restore CD8 + T cell effector function (25). Decabitine restore immune function and decreases mortality in sepsis (142).	Azacytidine, increases interferon responsiveness and antigen presentation in cancer (143–145).
***Histone Methylation***	EZH2/increases the methylation	3- deazaneplanocin (DZNeP).	DZNep (Inhibitor of SAM dependent enzymes), decreases methylation	H3K27me3, the repressive epigenetic mark induced by EZH2, persists to inhibit IL12 immunity at least 6 weeks after the initial septic insult (155). EZH2 expression increases proportional to disease severity and correlates with poor clinical outcomes in sepsis (156). In multiple cancers EZH2 induced epigenetic mark inhibit IFN-γ-JAK-STAT signaling (157–160). In TB, EZH2 is expressed early (161), with EZH2 inhibition decreasing *Ifng* and *Tnf* H3K27me2 and increasing TNF and IFN-γ production (162).	Inhibition of EZH2 with DZNep improved acute septic morbidity and mortality, lessen cytokine levels and bacterial burden in mice (163). EZH2 inhibition in TB decreases *Ifng* and *Tnf* H3K27me2 resulting in increased TNF and IFN-γ production (162).	GSK126, restores CD8 cell cytotoxicity (164). Combination of EZH2 and DNMT inhibitors synergistically increased IFN-γ responsiveness and CXCL9 and CXCL10 expression and shrinks tumor size during immunotherapy in ovarian cancer cell lines (165). EZH2 knockdown or pharmacologic inhibition restoring IFN-γ-induced gene expression in various cancers (157–160).
		GSK126	GSK126 (Selective inhibitor blocking EZH2), decrease methylation			

The PRC, which includes the HMT EZH2, interacts with DNMTs and also the NuRD complex. Therefore, these three suppressive epigenetic marks, H3K27 deacetylation, H3K27 methylation, and DNA methylation, often occur together, resulting in heterochromatin, thereby silencing gene expression (167). Chronic LCMV infection results in global decreased histone acetylation that limits both LCMV-specific and non-specific CD8 T cell effector function (147). Chronic LCMV infection also non-specifically decreases effector responses to influenza peptides, and decreases *salmonella* and *listeria* killing capacity (147, 168). Valproic acid, an HDAC inhibitor, was able to restore LCMV-specific and non-specific CD8 T cell effector function, including non-specific *listeria* killing capacity (147). Several studies indicate that HDAC inhibitors can restore host immunity when applied to chronic infections. Entinostat (MS-275), an inhibitor of HDAC1 and HDAC3, increases host anti-tumor immunity (152). Considering the long-term increased mortality that persists following a bout of sepsis, it would seem prudent to conduct a clinical trial to evaluate the efficacy of an HDAC inhibitor in reversing long-term sepsis-induced immune suppression (9).

Sirtuins, a class of HDACs, recognize the NAD^+^: NADH ratio and then deacetylate and silence NFĸB, TNF and IL1b after LPS-induced immune tolerance (20, 102). EX-527, a Sirt1 inhibitor restored myeloid cell IL1B and TNF production when administered after sepsis. Reinforcing the importance of timing, administering EX-527 early during sepsis increases mortality, however if given later during the immune hyporesponsive phase of sepsis, it reduces post-sepsis mortality in mice (112).

TB is the archetypical chronic infection. Macrophages infected with *Mtb* upregulate HDAC and undergo deacetylation of critical immune genes, such as IL12. Inhibition of HDAC restores immune function including cytokine production and *Mtb* killing capacity (148). In T cells, *Mtb*-induced upregulation of SIRT2 deacetylates NFĸB (p65) with SIRT2 inhibition improving both myeloid and lymphoid immunity, and *Mtb* killing capacity in mouse models (153). Sodium or phenyl butyrate, an HDAC inhibitor, restores IFN-γ downstream responsiveness (149, 150) as well as inflammasome and IL1 pathway gene expression (151). In a clinical trial that did not evaluate epigenetic or immunologic outcomes, the combination of Vitamin D_3_ and phenylbutyrate did not change time to sputum conversion but did ameliorate TB disease severity (151). Like sepsis, survivors of TB retain detrimental epigenetic scars (6, 22) and have increased all-cause mortality (12, 13). Large clinical trials should evaluate if reversing these detrimental epigenetic marks are able to reverse the post-infectious morbidity and mortality risk due to TB.

## Bioengineering Approaches to Reverse Epigenetic-Mediated Immune Exhaustion & Suppression

Systemic means to reverse immune suppression, such as immune checkpoint inhibitor blockade (e.g., anti-PD-1 and anti-LAG-3), have short and long-term toxicities (169). Newer technology such as the CRISPR/Cas9 system holds promise as a precise and controlled bioengineering tool (170–175) to reverse immune suppression. Typically, the CRISPR/Cas9 system includes a guide RNA (gRNA) complexed with the Cas9 protein to specifically edit a unique genomic address (170). For example, *in vitro* gene editing of the *PD-1* and *LAG-3* genes using CRISPR-Cas9 in CAR-T cells has improved their anti-tumor function (169, 176).

A catalytically inactive version of the Cas9 protein called dead, or deactivated, Cas9 (dCas9) (177) repurposes the CRISPR-Cas9 platform for precision edited of the epigenome or gene expression machinery (173–175, 177). A diverse spectrum of epigenetic effectors has been tethered to dCas9 to deliver epigenetic payloads to specific sites across the genome, giving rise to a continually expanding epigenome editing toolkit (173, 178). The Krüppel-associated box (KRAB) is a repressive domain that is a component of several zinc-finger transcription factors (179). A fusion protein between the KRAB domain and dCas9 (dCas9-KRAB) has been shown to promote highly specific gene silencing when targeted to mammalian genes (180) and to distal regulatory elements such as enhancers (181). A version of dCas9-KRAB with a linker for activation of T cells (LAT-dCas9-KRAB) was recently shown to silence the *PD-1* gene when targeted to its transcription start site (176).

Targeting the transcriptional start sites and promoters with dCas9 coupled with the *de novo* methyltransferases DNMT3A and its homolog DNMT3L (dCas9-DNMT3A/3L) has been described to produce widespread DNA methylation of CpG islands at the targeted loci for up to 1200 bp (182). In addition, tethering the catalytic domain of the DNA demethylase TET1 to dCas9 (dCas9-TET1) to promoters previously silenced by engineered transcriptional repressors can generate a stable, long-term reactivation of the silenced gene by demethylation of targeted CpG islands (183). Previous work has also shown that a fusion protein consisting of the catalytic core of the human acetyltransferase p300 and dCas9 (dCas9-p300) can achieve robust genetic transcriptional activation by targeting either promoters, proximal enhancers or distal enhancers (184).

The epigenome editing tools dCas9-TET1 and dCas9-p300 were recently employed to elucidate the epigenetic landscape of the *Foxp3* locus, an important transcription factor in T cells. Demethylation of the enhancer region of the *Foxp3* locus was achieved in mouse primary T cells, although without strong *Foxp3* gene expression. In contrast, targeting dCas9-p300 to the *Foxp3* promoter stabilized Foxp3 expression under both normal and inflammatory culture conditions *in vitro* (185). This technical approach provides new opportunities to revert anomalous post-infectious epigenetic modifications in other immunologically relevant genes using dCas9-based epigenome editing.

Robust targeted transcriptional activation has also been achieved by using CRISPR activation (CRISPRa) tools. dCas9 fused to an engineered tripartie activation domain consisting of VP64, p65 and Rta, (dCas9-VPR) has proven to be a potent synthetic CRISPR/Cas9-based transcriptional activator. For example, dCas9-VPR is able to induce gene activation of some target genes up to 320-fold compared to the original, conventional dCas9-VP64 activator (186).

Another method to increase transcriptional activation is by recruiting several copies of the regulatory proteins at once to the target gene. This can be achieved by fusing dCas9 to the SunTag, an array of a repeated short peptide sequence with strong affinity for a single-chain variable fragment (scFv) antibody fused to the activation domain. The SunTag can recruit up to 24 copies of the antibody-fused protein and has been used to recruit multiple copies of the transcriptional activation domain VP64, increasing gene expression of the targeted locus (187).

RNA aptamers that interact with transcriptional activation domains have been inserted into gRNAs, and these systems have been used to recruit transcriptional regulatory domains *via* dCas9 (188, 189). For example, the synergistic activation mediator consists of an MS2 bacteriophage coat protein-binding aptamer that is placed in the gRNA loops, which enables a fusion between MS2 p65 and Heat Shock Factor 1 (HSF1) to be successfully recruited to targeted genomic loci (188). Recently the SAM system was used to increase the expression of key endogenous genes related to immunological exhaustion in the context of boosting anticancer immunotherapy. Multiplexed gene activation of *Cd70*, *Cd80*, *Cd86*, *Ifnα4*, *Ifnβ1*, and *Ifnγ*  was achieved in mice using a CRISPRa gRNA library improving immunogenicity of the transduced cells and leading to tumor rejection *in vivo* (190).

Finally, Proteolysis Targeting Chimeras (PROTACs) are small molecules which induce the targeted degradation of a protein by linking it to an E3 ubiquitin ligase. The ubiquitinated protein is then recognized and degraded by the 26S proteasome (191). Recently, Si et al. demonstrated that a hematopoietic progenitor kinase has a key role in T cell exhaustion and could be targeted by employing PROTACs and CRISPR/Cas9 technology. Increased gene expression of the *MAP4K1* gene has been correlated with increased T cell exhaustion due to dysregulation of the NFκB signaling pathway. Knocking out this gene using a CRISPR/Cas9 system in CAR-T cells improved their persistence and functionality *in vivo*. Similarly, developing a small molecule PROTAC that selectively degrades the HPK1 protein encoded by the *MAP4K1* gene in CAR-T cells improves their efficacy as well (192).

A major challenge in reducing T cell exhaustion is the enduring epigenetic changes that differ from their normal state (25). The CRISPR/dCas9-based protein fusions to epigenetic writers and erasers are a potential tool to robustly and precisely modulate the epigenome of exhausted T cells, reverting them to their pre-infected functional state.

As previously discussed, T cell function requires balanced AP-1 and NFAT heterodimerization. CAR T cells experience tonic activation that induces characteristic features of exhaustion (193). By manipulating HA-28z CAR T cells to over-express c-Jun, AP-1-NFAT balance was restored, increasing IL-2 production (193). Recent studies have shown that the HDAC SIRT1 functions to deacetylate c-Jun, inactivating it, and thus effectively preventing the formation of the NFAT/AP-1 complexes required to induce *Il-2* expression in activated T cells. In this way SIRT1 acts as an epigenetic promoter of immune exhaustion (18, 194). In a follow-up CAR T study, the incorporation of a titratable FK506 binding protein 12 (FKBP) destabilizing domain (DD) emphasized the importance of timing and rest (195). Simply put, this engineered CAR T cell model demonstrated that interrupting tonic T cell activation, either through the titratable FK506 DD or through dasatinib, a tyrosine kinase inhibitor, could block epigenetic-mediated immune exhaustion.

## Conclusion

Increasing evidence from *in-vitro* studies and animal models has demonstrated the signaling pathways, TFs, metabolic intermediates and epigenetic enzymes that remodel chromatin in order to suppress gene expression and limit exuberant immune pathology. Although acutely, this suppression helps regulate an overly exuberant immune response, it makes individuals more susceptible to secondary infections and cancers leading to increased long-term morbidity and mortality. Other fields have harnessed drugs to manipulate epigenetic enzymes, metabolic pathways, TFs and signaling pathways to improve clinical outcomes. Similar studies need to evaluate which strategy limits off-target adverse effects in order to restore host immunity. For example, theoretically, upstream moderation of the three-metabolic-epigenetic-immune rheostats might better block detrimental epigenetic marks than a specific epigenetic modifying drug. Considering the significant long-term mortality that exists after pneumonia, sepsis and TB, translational studies using emerging immunologic approaches and bioengineering tools are needed to evaluate if modulating these pathways improve clinical outcomes.

## Author Contributions

All authors made a contribution to the acquisition of the information for the work, critically revised the manuscript for important intellectual content, and gave final approval of the current version to be published. All authors agree to be accountable for all aspects of the work in ensuring that questions related to the accuracy or integrity of any part of the work are appropriately investigated and resolved.

## Funding

AD is supported by NIAID K23 AI141681-02. AD, TN, RG-R, and IH are supported by the John S. Dunn Foundation. LS is supported by NIAID R01AI136831, R21 AI145539, P01AG051428 and OD P51 OD011133. CO is supported by STX- MSTP-NIH T32GM113896. IH is supported by CPRIT RR170030. RG-R is supported by Fulbright-García Robles Scholarship.

## Conflict of Interest

The authors declare that the research was conducted in the absence of any commercial or financial relationships that could be construed as a potential conflict of interest.
